# Focal cartilage defects of the lateral compartment do influence the outcome after high tibial valgus osteotomy

**DOI:** 10.1051/sicotj/2021044

**Published:** 2021-08-25

**Authors:** Tizian Heinz, Stephan Reppenhagen, Mike Wagenbrenner, Konstantin Horas, Malte Ohlmeier, Thomas Schäfer, Maximilian Rudert, Thomas Barthel, Manuel Weißenberger

**Affiliations:** 1 Department of Orthopaedic Surgery, University of Wuerzburg Koenig-Ludwig-Haus, Brettreichstr. 11 97074 Wuerzburg Germany; 2 Department of Orthopaedic Surgery, HELIOS ENDO-Klinik Hamburg 22767 Hamburg Germany

**Keywords:** Knee, Medial osteoarthritis, High tibial osteotomy, Cartilage defect

## Abstract

*Introduction*: High tibial medial open-wedge valgus osteotomy (HTO) is a well-established procedure for unicompartimental medial osteoarthritis of the young and active patient. However, the influence of cartilage defects of the lateral compartment on the total outcome remains obscure. *Methods*: From 2005 to 2012, a total of 63 patients underwent HTO for medial osteoarthritis of the knee at a single university orthopaedic center. Baseline data as well as intraoperative findings, including the grade and location of cartilage lesions, were evaluated retrospectively. Two groups were formed regarding the integrity of the lateral tibiofemoral compartment as measured by the Outerbridge score (group A: no lateral cartilage defects, group B: mild to moderate lateral cartilage defects). Functional outcome was assessed using the Knee and Osteoarthritis Outcome Score (KOOS), including its five subscores. *Results*: Comparing pre- and postoperative data, we identified an overall benefit of the HTO procedure as measured by the KOOS. Group A (no lateral cartilage defects) showed an increase in all five KOOS subscores (*p* = 0.00–0.01), whereas for group B (mild to moderate lateral cartilage defects), only two KOOS subscores revealed a significant increase (*p* = 0.03–0.04). There was also a statistically significant difference in the total KOOS score with higher values for group A at the postoperative visit. Cartilage defects with a higher Outerbridge score were associated with lower postoperative KOOS subscores. *Discussion*: Mild to moderate cartilage defects of the lateral compartment humble the total outcome after HTO procedure. Thus, indication for HTO should be made very carefully if any degree of lateral cartilage degeneration is present.

## Introduction

Osteoarthritis (OA) is one of the most common musculoskeletal disorders, especially among the elderly population. Optimal treatment strategies, especially for younger patients, are scarce. Moreover, the available strategies are controversially discussed in current literature. In Germany, the total 12-months prevalence of osteoarthritis has been reported to be 17.9% of adults over 18 years. A steep increase by age was recognized, reaching as high as 48.1% of affected women aged over 65 years [1]. Risk factors for osteoarthritis can be divided into non-modifiable and modifiable factors, with malalignment of the lower extremity being assigned to the latter. Various studies have demonstrated that malalignment can cause increased stress across a focal joint area and thus leading to subsequent cartilage loss and osteoarthritis [2–4]. Even in a neutrally aligned limb, the force distribution across the knee joint is not completely equally distributed between the medial and lateral compartment. In particular, 60–70% of the force is directed to the medial compartment during weight-bearing activities. This may explain the higher prevalence of the medially emphasized osteoarthritis of the knee compared to lateral osteoarthritis [5–9]. In a varus aligned knee, the load-bearing mechanical axis is shifted to the medial compartment leading to a 7.4-fold increased body weight force across this joint area during walking and a fourfold increased risk of progression of medial OA [10, 11]. While there is a broad consensus for the use of total knee arthroplasty (TKA) in late-stage, symptomatic bicompartmental knee OA amongst orthopaedic surgeons, operative treatment strategies for unicompartmental medial OA remain controversial. Both unicompartmental knee arthroplasty (UCA) and high tibial valgus osteotomy (HTO) are recognized and well-accepted treatment options for medial OA of the knee [12–15]. However, the right and thoughtful patient assignment to the aforementioned procedures represents a critical step to gain favorable and satisfactory outcome parameters. This study aimed to assess the functional outcome as measured by the Knee and Osteoarthritis Outcome Score (KOOS) after HTO for osteoarthritis of the medial compartment in varus aligned knees was performed. Special interest was put on how cartilage defects of the lateral compartment impact the total postoperative outcome score. We hypothesized that even with mild to moderate cartilage defects of the lateral compartment expectations on HTO should be humbled.

## Materials and methods

### Patient recruitment and study population

A retrospective study of patients undergoing HTO due to varus deformity of the proximal tibia was performed. Data analysis was performed using an electronic medical report system. From August 2005 to March 2012, a total of 63 patients were included in this study. Inclusion criteria were an ongoing complaint of knee pain, osteoarthritis of the medial compartment with a Kellgren–Lawrence-Score of not more than three or a focal cartilage lesion of the medial compartment, patient age under 60 years, and knee flexion of at least 120°. High-grade ligamentous instabilities, as defined by simultaneously present coronal and sagittal laxity of the knee, as well as inflammatory arthropathy, were defined as exclusion criteria. Furthermore, mild to moderate patellar lesions (Outerbridge ≥ 2) lead to exclusion from the study. In every case, a weight-bearing anteroposterior long-axis view was obtained before surgery to determine the preoperative knee alignment. Calculation of the planned correction osteotomy was made electronically using planning software (mediCAD, Version 3.0, Hectec GmbH, Germany). Six weeks postoperatively, another weight-bearing anteroposterior long-axis view was obtained. From 2005 to 2012, a total of 63 patients underwent HTO by meeting the aforementioned criteria in a single orthopaedic center. In every case, a medial open-wedge approach was used and a Tomofix^®^ plate secured the osteotomy. Written informed consent for participation was obtained from every individual and the study had been approved by the ethics committee of The University of Wuerzburg.

### Surgical treatment and postoperative rehabilitation

Diagnostic arthroscopy of the knee joint was routinely performed before HTO to evaluate the current state of cartilage degeneration of the medial and lateral compartment. Further, the degree of meniscal degeneration and ligamentous injuries were assessed. Meniscal injuries detected during diagnostic arthroscopy were addressed via a partial resection of the injured pattern. The same applied to areas of degenerated cartilage which were addressed via debridement of the affected area. Cartilage lesions were classified according to the Outerbridge classification system. Both for the medial and lateral compartment, a total Outerbridge score was calculated by summarizing the highest cartilage defect of the femoral condyle and the corresponding tibial plateau of one compartment. Thus, a score ranging from 0 to 8 could be formed for each compartment, with a score of 8 indicating a progressive cartilage degeneration of one compartment. Therefore, the authors considered a score of one to three as a mild cartilage degeneration. Accordingly, score values between four and six were considered as moderate cartilage defects of the medial or lateral compartment. Score values higher than six were defined as progressive cartilage defects. For better comparability, two groups were formed afterwards: group A with no cartilage defects (total lateral Outerbridge score 0) and group B with mild to moderate cartilage degeneration of the lateral compartment (total lateral Outerbridge score one to six). After the diagnostic arthroscopy, the HTO procedure followed subsequently. Therefore, the medial proximal tibia was exposed by a longitudinal incision. A biplanar osteotomy was then performed by an ascending cut starting from the medial border of the tibia towards the tibiofibular joint followed by a subsequent tuberosity cut parallel to the anterior tibial margin. The osteotomy was opened gradually using chisels and a spreading device. After the desired amount of tibial opening has been achieved, the osteotomy was secured by a Tomofix^®^ plate. The aim of correction was defined as a slight valgus of 3–5° relative to the mechanical axis defined by Dugdale et al. and thus unloading the medial compartment [16]. Postoperatively, all patients underwent a standardized rehabilitation protocol. Continuous passive motion without resistance was recommended from day one postoperatively for six weeks without weight-bearing of the treated extremity. Weight-bearing anteroposterior long-axis views were obtained six weeks postoperatively, and if the bone healing was considered sufficiently, a stepwise increase until full weight bearing from 9 to 10 weeks postoperatively was achieved.

### Follow-up evaluation

The German version of the Knee and Osteoarthritis Outcome Score (KOOS) was used to assess the surgical outcome after HTO was performed. The KOOS has been validated for various pathologies of the knee, including OA [17]. The German version of the KOOS has also been validated for measuring therapeutic effects of knee-related conditions [18]. The KOOS questionnaires were handled out to the patients postally, and both the pre-and postoperative condition was evaluated retrospectively.

### Statistical analysis

Data management and analysis were conducted using IBM SPSS Statistics version 26 (IBM Corp., Armonk, NY, USA). Baseline variables and the KOOS outcome score were assessed using standard descriptive statistics. For continuous variables, means and standard deviations were calculated, whereas absolute and relative frequencies are shown for categorial variables. Data were checked for normal distribution using the Kolmogorov–Smirnov test and Shapiro–Wilk test. *P*-values < 0.05 were considered statistically significant. For normally distributed data, comparisons were made using Student’s *T*-test. For not normally distributed data, the Mann–Whitney–*U* test and Kruskal–Wallis test were performed. Correlations were identified based on the Pearson correlation coefficient, respectively, the Spearman’s correlation coefficient for metrically and ordinally scaled variables.

## Results

### Patient demographics

In the whole study cohort, the mean patient age was 39 years (range from 14 to 58 years), and the mean body-mass-index (BMI) was 27 kg/m^2^ (17–40 kg/m^2^). The left and right extremity were nearly equally affected (51% vs. 49%). Of the 63 patients included in the study, 54 were males and 9 were females. Details regarding demographic and preoperative findings, specifically of the two considered study groups, are depicted in Table 1. Fifteen patients (22%) stated to have a regulatory intake of nicotine of at least three cigarettes per day. The mean Kellgren–Lawrence-Score was 2.3 ± 0.6 for the medial compartment and 1.3 ± 0.5 for the lateral compartment, respectively. The mean Outerbridge score for the medial compartment was significantly higher than the lateral compartment (4.8 ± 2.5 vs. 2.5 ± 1.2, *p* < 0.00). There was a statistical difference in the Kellgren–Lawrence-Score of the medial compartment between both groups (*p* = 0.01) (Table 1). Bivariate correlation analysis revealed a poor correlation of the Kellgren–Lawrence-Score of the medial compartment with the Outerbridge-Score of the lateral compartment (*r* = 0.33, *p* = 0.01). The mean anatomical medial proximal tibia angle (aMPTA) before surgery was 84.2° ± 2.6 (range 75.4–88.7) compared to 92.5° ± 3.3 (range 84.9–98.9) in the postoperative radiographs. Preoperative values of the aMPTA below 88.0 were considered a pathological tibial varus deformity [19]. The mean follow-up period averaged 46.1 ± 24.3 months (10.0–93.0 months). The medial wedge was left unfilled or filled using autologous spongiosa or bone graft substitute (Table 2). Details on the mean Outerbridge score of the medial and lateral femorotibial compartment are given in Table 3.

**Table 1:** Demographic and surgical data of Group A and Group B at the time of surgery.

	Group A (no lateral cartilage lesions)	Group B (mild to moderate lateral cartilage lesions)	P-value for statistical difference
Age (years)	39.2 ± 12.9	45.2 ± 7.9	0.06
BMI (kg/m²)	25.6 ± 4.9	27.9 ± 4.3	0.09
Preoperative varus deformitiy (in °)	7.7 ± 2.3	6.5 ± 3.3	0.17
Kellgren–Lawrence medial compartment	2.2 ± 0.5	2.6 ± 0.7	0.01*
Kellgren–Lawrence lateral compartment	1.3 ± 0.5	1.4 ± 0.6	0.49
Ratio male : female patients	34 : 5	20 : 4	–
Outerbridge score lateral femoral condyle	–	1.1 ± 0.7	–
Outerbridge score lateral tibial plateau	–	1.4 ± 0.6	–
aMPTA, preoperative (in °)	84.0 ± 2.4	84.4 ± 3.1	0.57
aMPTA, postoperative (in °)	92.4 ± 3.4	92.0 ± 3.5	0.69

Significant differences are highlighted by an asterisk. aMPTA: anatomical medial proximal tibial angle.

**Table 2:** The medial wedge was left unfilled or was reconstructed using bone substitute.

Wedge interposition	Total number (*n*)	Relative number (%)
Autologous spongiosa	25	40
ChroNos®	31	49
None	7	11

**Table 3:** Details on the mean Outerbridge score of the medial and lateral femorotibial compartment.

Area of cartilage defect	Mean Outerbridge score ± SD	Range
Lateral femoral condyle	0.4 ± 0.7	0–3
Lateral tibia	0.5 ± 0.8	0–3
Lateral compartment	0.9 ± 1.4	0–6
Medial femoral condyle	2.5 ± 1.3	0–4
Medial tibia	2.4 ± 1.4	0–4
Medial compartment	4.8 ± 2.5	0–8

### Meniscal and ligamentous integrity

Nearly one-third of the patients showed to have an intact medial meniscus at the time of the surgery. Seventy percent had either a stable partially resected medial meniscus due to arthroscopic surgery before the HTO procedure or showed to have a meniscal lesion requiring partial meniscectomy at the time of the HTO procedure (Table 4). Regarding the lateral meniscus, there was only one case with an injury of the lateral meniscus that was treated by partial meniscectomy at the time of the HTO procedure. Coronal stability of the knee was given in all 63 cases by both intact medial and lateral collateral ligaments. Only two patients revealed a remarkedly sagittal instability by a total rupture of the anterior cruciate ligament (ACL) that was addressed using an autologous graft (semitendinosus tendon) at the time of the HTO procedure. Another two patients underwent ACL reconstruction surgery using an autologous graft at least six months before the HTO procedure. Still, they showed sagittal stability at the time of the HTO procedure.

**Table 4:** Details on the meniscal and ligamentous integrity of the affected knee at the time of the HTO procedure.

Meniscal and ligamentous integrity of the knee	Total number (*n*)	Relative number (%)
Medial compartment		
Intact meniscus	18	29
Meniscal lesion	13	20
Intact meniscal stump (partially resected)	32	51
Intercondylar region		
Intact ACL	59	94
Ruptured ACL	2	3
ACL-graft	2	3
Intact PCL	63	100
Ruptured PCL	0	0
PCL-graft	0	0
Lateral compartment		
Intact meniscus	62	98
Meniscal lesion	1	2
Intact meniscal stump (partially resected)	0	0

### Functional outcome and KOOS

**Figure 1:**
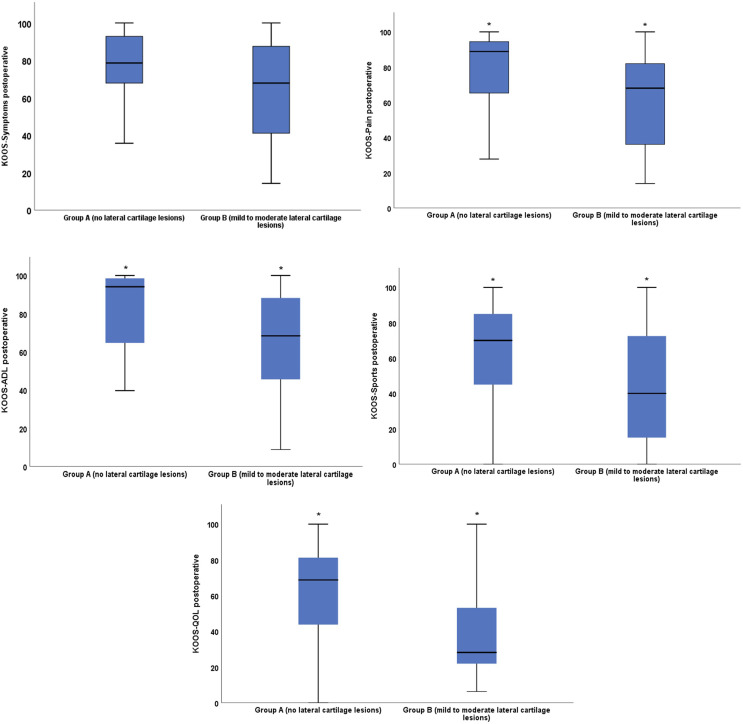
KOOS subscores at the postoperative visit for Group A and Group B.

A significant improvement in all five KOOS subscale scores during the follow-up could be observed (*p* < 0.00). A bivariate correlation analysis revealed a significant negative correlation between the postoperative KOOS subscores and the total Outerbridge score of the lateral compartment. However, this did not apply to the KOOS Symptoms subscale. There were significantly lower (i.e., worse) KOOS subscales scores for patients with lateral cartilage defects (group B) compared to patients with no cartilage defects of the lateral compartment (group A) (Figure 1). From the preoperative to the postoperative visit, group A showed a significant increase in five KOOS subscale scores, whereas for group B a significant increase could be observed only for the KOOS-ADL (Activity of daily life) and KOOS-Pain score (Table 5). Regarding the preoperative KOOS subscores, no significant differences could be observed between both groups (Table 6). No statistically significant difference for both groups could be shown at the preoperative visit regarding the total KOOS score. However, at the postoperative visit, values of the total KOOS score were significantly higher for group A (Table 6).

**Table 5:** Changes of the KOOS subscores from the pre- to the postoperative visit for Group A and B.

Group A (no lateral cartilage lesions, *n* = 39)			Group B (mild to moderate cartilage lesions, *n* = 24)	
KOOS subscore	Mean increase from pre- to postoperative visit (±SD)	*P*-value for statistical significance	Mean increase from pre- to postoperative visit (±SD)	*P*-value for statistical significance
KOOS-Symptoms	12.6 ± 24.2	0.00*	9.8 ± 22.2	0.10
KOOS-Pain	24.6 ± 25.7	0.00*	20.5 ± 34.0	0.03*
KOOS-ADL	21.8 ± 24.1	0.00*	19.5 ± 34.2	0.04*
KOOS-Sports	20.3 ± 33.5	0.00*	10.3 ± 38.8	0.31
KOOS-QOL	25.8 ± 25.4	0.00*	12.5 ± 30.4	0.12

Significant differences are highlighted by an asterisk. SD: Standard deviation.

**Table 6:** Mean pre- and postoperative KOOS subscores and statistical differences for Group A and Group B.

KOOS subscore	Mean KOOS score (± SD), Group A (no lateral cartilage lesions)	Mean KOOS score (± SD), Group B (mild to moderate cartilage lesions)	*P*-value for statistical difference
Preoperative			
KOOS-Symptoms	62.8 ± 27.9	55.2 ± 28.3	0.42
KOOS-Pain	53.6 ± 27.8	42.9 ± 26.6	0.26
KOOS-ADL	60.2 ± 32.4	48.9 ± 29.7	0.29
KOOS-Sports	41.0 ± 36.5	36.5 ± 41.6	0.73
KOOS-QOL	31.3 ± 27.8	26.9 ± 25.9	0.64
KOOS-Total	51.7 ± 28.7	39.5 ± 25.1	0.17
Postoperative			
KOOS-Symptoms	78.3 ± 17.4	62.6 ± 27.4	0.03*
KOOS-Pain	81.1 ± 19.1	58.1 ± 28.4	0.00*
KOOS-ADL	83.8 ± 19.4	62.9 ± 27.1	0.01*
KOOS-Sports	65.2 ± 29.4	41.2 ± 32.3	0.02*
KOOS-QOL	59.3 ± 25.6	34.6 ± 25.3	0.01*
KOOS-Total	73.8 ± 20.3	54.1 ± 24.2	0.01*

Statistically significant values are marked by an asterisk. SD: standard deviation.

### Pre- and postoperative alignment

This study cohort’s mean preoperative mechanical femorotibial angle was 172.9° ± 2.7° of varus angulation. After the HTO procedure, the postoperative mean femorotibial angle was 181.4° ± 2.6° of valgus. Preoperatively planned and postoperatively achieved femorotibial valgus angle did not differ significantly (182.0° ± 1.1° vs. 181.4° ± 2.6°, *p* = 0.1). The postoperative Mikulicz-line passed the tibial plateau at a mean width of 54.7% ± 12.2%, defined as the target area defined by Fujisawa [20]. However, a bivariate correlation analysis showed no significant correlation between the postoperative KOOS subscores and the postoperative femorotibial valgus angle (*r* = −0.1 to 0.60, *p* = 0.4–0.8). The same applied to the tibial width of the postoperatively crossing Mikulicz-line: no correlation could be found with the postoperative KOOS subscores (*r* = 0.1, *p* = 0.4–0.7).

### Complications

Overall, complications were rare. There were three cases of which complications were documented. Two patients suffered from postoperative wound infections that could be successfully addressed solely by the prolonged administration of antibiotics without the need for operative revision surgery. In one case, a prolonged bone healing process was addressed properly by a prolonged period of partial weight-bearing. No case of a recurring postoperative varus alignment of the treated extremity has been documented. In two cases, conversion to TKA has been documented, the first after 6.2 years and the second after 8.5 years after the index procedure.

## Discussion

Recent research has shown that the HTO procedure can significantly reduce pain and improve knee function in patients with medial osteoarthritis by delaying the progress of degeneration of the medial compartment [4]. The HTO procedure leads to unloading of the medial compartment by shifting the weight-bearing axis towards a more lateralized one. By a four-degree mechanical femorotibial valgus angle, the load-bearing is assumed to be distributed equally between the medial and lateral compartment [21]. As such, great consensus exists between orthopaedic surgeons to aim for a slightly postoperative valgus alignment of the knee. Many authors suggest a postoperative femorotibial valgus angle of 5–10° which equates the intersection of the weight-bearing line at 62–66% of the tibial width [21–25]. The ideal correction angle is believed to allow for sufficient unloading and regeneration of the medial compartment while at the same time avoiding overloading and degeneration of the lateral compartment [26–28].

Generally, the influence of the HTO procedure on laterally located cartilage defects of the knee remains controversial [29–31]. While a high-grade medial cartilage degeneration is generally believed to negatively impact the outcome, little is known about the lateral compartment’s natural course of cartilage defects when performing HTO [32, 33]. For this reason, a special interest of this study focused on whether patients with asymptomatic mild to moderate cartilage defects of the lateral compartment are likewise expected to benefit from HTO. As such, the aim was to investigate whether these patients can achieve comparable results to patients with normal lateral knee compartments and thus can be regarded as equally indicated for HTO. Some studies suggest that asymptomatic mild to moderate focal cartilage defects of the lateral compartment can be very well accepted without negatively influencing the total benefit of the HTO procedure [34, 35].

However, from the data of the present study, a conclusion contradictory to this assumption can be drawn. Deducing from the results of this retrospective analysis, mild to moderate cartilage degeneration of the lateral compartment leads to significantly worse postoperative outcome scores than those with intact lateral knee compartments. This major finding is partially in concordance with a recent study from Hohloch et al. showing that cartilage defects of the lateral compartment are generally associated with lower outcome scores. However, statistical significance for this finding was lacking [29]. A recent study from Jin et al. concluded that grade ≥ 2 cartilage defects of the lateral compartment feature a significant risk factor for failure of the HTO procedure [36].

These findings seem to be reasonable to some point: With increasing load-bearing of the lateral compartment, prior asymptomatic cartilage lesions likely tend to get symptomatic and may even tend towards progression [37]. From the data of this study, it can be hypothesized that even a meniscus intact lateral knee compartment at the time of surgery seems to be unable to fully compensate for the increased weight-bearing of the cartilage defects and thus not preventing them from getting symptomatic. This may be unproblematic if the cartilage of the lateral compartment shows normal integrity. An animal-based study of Ziegler et al. could demonstrate that neither macroscopic nor microscopic changes of the lateral tibiofemoral compartment can be found at six months after HTO [31]. However, it is stated that at least some progressive changes of the lateral meniscus after the HTO procedure can be observed [30, 38].

Moreover, concerns and criticism regarding the routinely performed arthroscopy before the HTO procedure have been raised recently, as some studies failed to show any relevance of the arthroscopic findings with the total outcome of the HTO procedure [39, 40]. Taking the present study’s findings into account, the principle of a routinely performed arthroscopy prior to an osteotomy around the knee can be strongly encouraged. The presence of any focal lateral cartilage defects should then lead to a thorough re-evaluation of the planned osteotomy based on the findings of this study. Regarding minor findings of this study, the authors could demonstrate a negative bivariate correlation between the postoperative KOOS outcome score and the total degree of lateral cartilage degeneration according to the Outerbridge classification system. This suggests that a worse outcome with HTO can be expected with a higher degree of focal lateral cartilage defects. The postoperative valgus angle did not reveal any correlation with the final functional outcome scores, which concordance with existing literature [29, 41].

We are aware that the current study has several limitations. As this research was designed as a retrospective analysis, the level of evidence should generally be considered inferior to prospective cohort studies, and results may be biased. There was a statistically significant difference in the Kellgren–Lawrence Score of the medial compartment between both groups before surgery which might serve as a resource for some bias. However, this difference may be elicited by the relatively poor intra- and interrater variability of the Kellgren–Lawrence-Score [42, 43]. Especially the scoring between the second and third grade of the Kellgren-Lawrence-Score seems to warrant difficulties by the insufficient interrater variability in assessing the joint space narrowing on plain radiographs [14]. Yet the study’s patient number and follow-up period seemed to be adequate despite a relatively large time span of the follow-up data acquisition [29, 41]. A further limitation is the availability of only one outcome score that was obtained pre-and postoperatively. Nonetheless, the KOOS is a powerful and validated tool that has also been validated for measurements of therapeutic effects of knee-related conditions [18].

## Conflict of interest

The authors declare that they have no relevant financial or non-financial interests to report.

## Funding

This research did not receive any specific funding.

## Ethical approval

The ethics committee of the University of Wuerzburg has approved the retrospective study of this article. Written informed consent for participation was obtained from every individual.

## Informed consent

Written and informed consent was obtained from every individual.

## Authors contributions

*T. Heinz*: Writing, Conceptualization, Drafting, Statistical analysis. *S. Reppenhagen*: Writing, Conceptualization, Methodology. *M. Wagenbrenner*: Statistical analysis, Writing, Visualization, Reviewing. *K. Horas*: Writing, Drafting, Investigation. *M. Ohlmeier*: Writing, Editing, Investigation, statistical analysis. *T. Schäfer*: Writing, Drafting, Editing, Formatting. *M. Rudert*: Writing, Reviewing, Supervision, Editing. *T. Barthel*: Writing, Methodology, Reviewing, Visualization. *M. Weißenberger*: Supervision, Writing, Reviewing, Editing, Conceptualization, Validation.
